# Caspase-2 is a mediator of apoptotic signaling in response to gemtuzumab ozogamicin in acute myeloid leukemia

**DOI:** 10.1038/s41420-022-01071-9

**Published:** 2022-06-11

**Authors:** Petra Hååg, Magnus Olsson, Jeremy Forsberg, Marita Lagergren Lindberg, Bo Stenerlöw, Dali Zong, Lena Kanter, Rolf Lewensohn, Kristina Viktorsson, Boris Zhivotovsky, Leif Stenke

**Affiliations:** 1 Department of Oncology-Pathology, Karolinska Institutet, SE-171 64 Solna, Sweden; 2 Institute of Environmental Medicine, Karolinska Institutet, SE-171 77 Stockholm, Sweden; 3 Department of Immunology, Genetics and Pathology, Rudbeck Laboratory, Uppsala University, SE-75185 Uppsala, Sweden; 4 Theme Cancer, Medical Unit head and neck, lung and skin tumors, Thoracic Oncoflogy Center, Karolinska University Hospital, SE-171 64 Solna, Sweden; 5 Faculty of Medicine, Lomonosov Moscow State University, 191992 Moscow, Russia; 6 Theme Cancer, Department of Hematology, Karolinska University Hospital, Stockholm, Sweden; 7 Department of Medicine Solna, Karolinska Institutet, SE-171 64 Solna, Sweden; 8 Present Address: Laboratory of Genome Integrity, National Cancer Institute, National Institute of Health, Bethesda, MD USA

**Keywords:** Cancer, Preclinical research

## Abstract

The antibody conjugate gemtuzumab ozogamicin (GO; Mylotarg^®^) provides targeted therapy of acute myeloid leukemia (AML), with recent approvals for patients with CD33-positive disease at diagnosis or relapse, as monotherapy or combined with chemotherapeutics. While its clinical efficacy is well documented, the molecular routes by which GO induces AML cell death warrant further analyses. We have earlier reported that this process is initiated via mitochondria-mediated caspase activation. Here we provide additional data, focusing on the involvement of caspase-2 in this mechanism. We show that this enzyme plays an important role in triggering apoptotic death of human AML cells after exposure to GO or its active moiety calicheamicin. Accordingly, the caspase-2 inhibitor z-VDVAD-fmk reduced GO-induced caspase-3 activation. This finding was validated with shRNA and siRNA targeting caspase-2, resulting in reduced caspase-3 activation and cleavage of poly [ADP-ribose] polymerase 1 (PARP-1). We previously demonstrated that GO-induced apoptosis included a conformational change of Bax into a pro-apoptotic state. Present data reveal that GO-treatment also induced Bid cleavage, which was partially reduced by caspase-2 specific inhibition while the effect on GO-induced Bax conformational change remained unaltered. In mononuclear cells isolated from AML patients that responded to GO treatment in vitro, processing of caspase-2 was evident, whereas in cells from an AML patient refractory to treatment no such processing was seen. When assessing diagnostic samples from 22 AML patients, who all entered complete remission (CR) following anthracycline-based induction therapy, and comparing patients with long versus those with short CR duration no significant differences in baseline caspase-2 or caspase-3 full-length protein expression levels were found. In summary, we demonstrate that GO triggers caspase-2 cleavage in human AML cells and that the subsequent apoptosis of these cells in part relies on caspase-2. These findings may have future clinical implications.

## Introduction

Acute myeloid leukemia (AML) is the predominant leukemia in adults. Despite multimodal chemotherapy, poor outcome is still common with long-term survival only seen in approximately 20% of the patients [1]. Although regular high-dose chemotherapy (CT), typically comprised of an anthracycline (e.g. daunorubicin) and cytarabine (ara-C), frequently induces complete remission (CR), most of these patients relapse with a chemoresistant phenotype [2]. Alternative targeted therapy approaches involve the use of monoclonal antibodies, either engineered or conjugated to small molecules or radionuclides [3]. Gemtuzumab ozogamicin (GO; Mylotarg^®^) was the first targeted antibody-drug conjugate (ADC) for AML approved by the U.S. Food and Drug Association (FDA) [2, 4, 5]. It comprises a humanized IgG4 antibody (hP67.6), directed against the surface antigen CD33 and a conjugate of calicheamicin, γ_1_ (N-acetyl gamma calicheamicin γ_1_ dimethyl hydrazide) [4, 5]. The use of the CD33 transmembrane glycoprotein for targeted therapy delivery in AML was prompted by earlier studies showing higher CD33 expression level in AML leukemic blasts, as compared to normal myeloid cells [4]. Thus, most of the AML patients have at least a proportion of CD33-expressing blasts, albeit the magnitude varies considerably. The CD33 expression level appears linked to the differentiation status of the leukemia clone (e.g., high level is seen in acute promyelocytic leukemia; APL), the cytogenic profiles and to certain mutations, such as *NPM1* and *FLT3/ITD* [4]. Given this, GO offers a way to more specifically target leukemic blasts [2, 4]. GO was, when granted accelerated approval for relapsed AML by the FDA in 2000, used as single agent and provided second remission rates of approximately 25% in aggregate phase 2 studies [6]. When subsequently used in combination with daunorubicin and ara-C (DA), GO-induced toxicity, in particular adverse liver events, raised concerns, leading to the withdrawal of the FDA approval in 2010 [7]. However, additional phase 3 studies combining lower doses of GO with DA showed improved event-free and overall survival, particularly in patients with favorable- and intermediate-risk AML karyotypes [8, 9]. These data motivated FDA to renew their approval for GO in September 2017, this time for CD33-positive AML both in front line and in relapsed settings [10]. The following year GO was also approved by the European Medicines Agency (EMA) to be used in Europe in adult patients with newly diagnosed CD33^+^ AML [11]. GO is also approved in the US to be used in combination with all-*trans* retinoic acid (ATRA) and arsenic trioxide (ATO) [10], and as standard treatment for core-binding factor positive AML (CBF-AML) [10, 11]. Thus, the clinical efficacy and usefulness of GO is obvious in CD33-positive AML, but the more precise molecular mechanisms behind GOs ability to selectively kill AML blasts remain to be further elucidated. So far, the mechanisms involve binding of GO to the CD33 antigen followed by cellular uptake of the formed complex and redistribution from endosomes to lysosomes where the calicheamicin moiety is set free from the CD33-antibody carrier via cleavage. The free calicheamicin subsequently intercalate with DNA in the nucleus resulting in the formation of DNA double-strand breaks (DSBs), induction of the cell cycle arrest and activation of cell death [4, 5]. Thus, we and others previously reported that GO-induced cell death in part involves activation of apoptosis via the intrinsic mitochondrial route, leading to caspase-3 activation and cleavage of its substrates [12, 13].

In this work we focused on caspase-2 and its role in GO-induced apoptotic cell death. Caspase-2 has been shown to be important for the initiation and execution of apoptosis in response to DNA damaging drugs, e.g., cisplatin, etoposide, and doxorubicin [14–18]. Caspase-2 activation involves the dimerization of inactive monomers in multimeric protein complexes, which may be executed in a PIDDosome (p53-induced protein with a death domain (PIDD) and RIP-associated ICH-1/CAD-3 homologous protein with a death domain (RAIDD))-dependent or independent manner [19–23]. It was also reported that DNA damage triggers caspase-2 activation in the nucleolus, where the activation involves not only PIDD and RAIDD but also nucleophosmin (NPM1) [24]. Mutation in *NPM1* (NPM1c^+^) is prevalent in AML and impairs protein function as a result of the nucleus to cytoplasm protein dislocation [25–27]. Moreover, it was demonstrated that NPM1c^+^ AML cells fail to induce caspase-2 cleavage after DNA damaging treatment [24].

Here we report that GO- and calicheamicin-induced apoptotic cell death in AML cells in vitro involves the processing of caspase-2 to its active form. We show that inhibition of caspase-2 catalytic activity blocks GO-induced caspase-3 activation and reduces cleavage of full-length Bid while having no effect on GO-induced conformational change of Bax into a pro-apoptotic state. In line with a role for caspase-2 in GO-induced apoptosis, siRNA- or shRNA-mediated knockdown of caspase-2 expression reduced caspase-3 processing and PARP-1 cleavage following treatment with GO or calicheamicin. In AML patient-derived blasts treated in vitro with GO, full-length caspase-2 protein level was decreased in GO-sensitive ones, indicating processing, while still being present in refractory cells. However, neither basal caspase-2 nor caspase-3 full-length expressions in AML patient cells was linked to CR duration upon anthracycline-based induction therapy.

## Results

### GO triggers caspase-2 processing

**Fig. 1:**
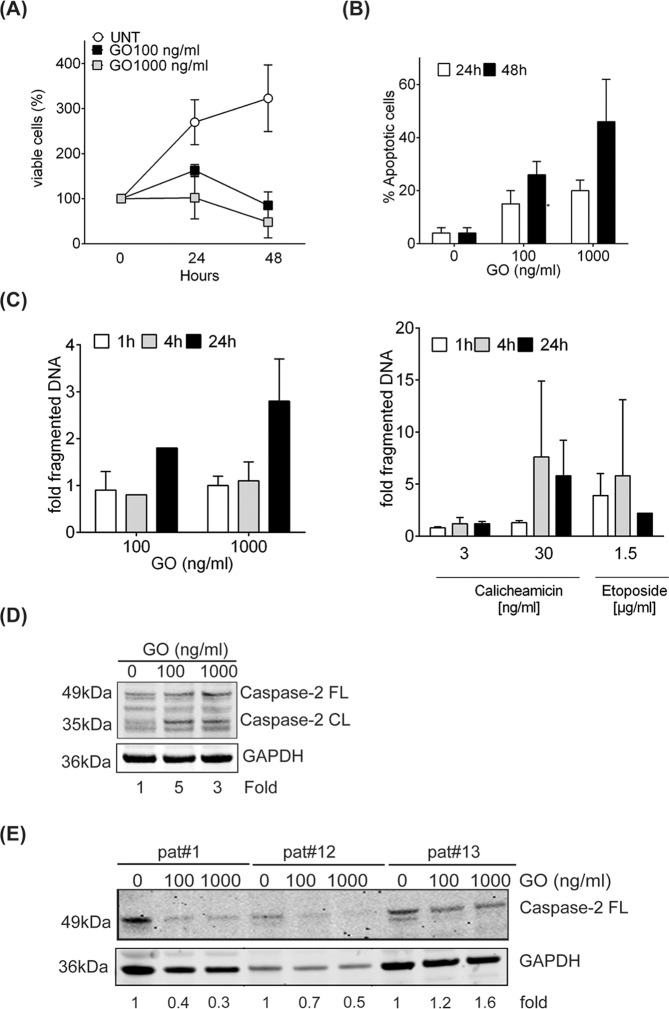
GO-induced apoptotic signaling involves caspase-2 processing. **A** HL60 AML cells were treated with GO 100 ng/ml or 1000 ng/ml for 24 h or 48 h. Cell viability was assessed by trypan blue exclusion staining. Data shown is the average of three independent experiments with bars representing SD. **B** Apoptotic associated fragmentation of cell nuclei was analyzed in samples treated as in (**A**) by staining with DAPI and counting in fluorescence microscope. Data shown is the percentage of cells showing apoptotic fragmentation of DNA in nucleus out of 200 cells counted. Data given is the mean of three biological replicates with bars representing SD. **C** DNA DSB rejoining was assessed in HL60 cells at indicated time points after treatment with GO (*left panel*), calicheamicin or etoposide (*right panel*) using PFGE. The amount of DNA DSBs is presented as fold fragmented DNA (size < 5.7 Mbp) relative to untreated cells. **D** Full-length and processed caspase-2 (51 kDa and 35 kDa, respectively) were analyzed in HL60 cells following GO treatment for 24 h. Fold calculation were comparing the 35 kDa cleaved fragment in relation to untreated cell after adjustment to GAPDH expression. Figure shown is representative of two independent biological replicates. **E** Full-length caspase-2 expression was analyzed by western blot in primary mononuclear cells from AML patients (pat#1, #12, and #13; Table 1) treated ex vivo for 24 h with indicated GO doses. Expression of full-length caspase-2 was quantified in relation to each patient untreated sample set to 1 after normalization to the GAPDH loading control.

We and others previously reported that GO-induced apoptosis in AML cells involves caspase-3 activation [12, 13]. In this study, we analyzed if caspase-2 played a role in GO-induced apoptotic signaling. In line with previous reports [12, 13], treatment with GO caused a clear time- and dose-dependent inhibition of cell proliferation in HL60 AML cells (Fig. 1A). GO also triggered induction of apoptotic morphology, starting at 24 h with a further increase at 48 h post-treatment (Fig. 1B). Next, we analyzed if these doses of GO also caused induction of DNA DSBs in HL60 cells with calicheamicin and etoposide used as comparative agents (Fig. 1C). As seen, GO induced an about two- to three-fold increase in DNA DBS 24 h post-treatment when 100 ng/ml or 1000 ng/ml GO were applied while at 4 h post-treatment no clear increase was evident (Fig. 1C, left panel). In contrast, calicheamicin and etoposide both induced a faster increase of DNA DSBs starting already 1 h and 4 h post-treatment, respectively (Fig. 1C).

Activation of apoptosis following DNA damage may be executed via mitochondria-dependent or mitochondria-independent routes, which in part involves caspase-2 proteolytic activity [14–17, 21, 24, 28–31]. Given that calicheamicin, the cytotoxic payload of GO, triggers DNA DSBs formation as part of its action mechanism [32], we next examined if caspase-2 processing occurred during GO treatment. The amount of the 35 kDa cleaved form of caspase-2 was increased 3–5-fold compared to untreated cells after 24 h treatment, indicative of caspase-2 processing and protease activation (Fig. 1D). To analyze whether processing of caspase-2 also occurred in primary AML cells, caspase-2 expression was investigated in mononuclear cells derived from three individual AML patients treated with GO in vitro (Fig. 1E). Western blot analysis indicated that GO-treatment resulted in the processing of caspase-2, as visualized by a decrease in GAPDH normalized expression of the full-length caspase-2 protein by about 50% in two out of three patient samples (Fig. 1E).

### Caspase-2 inhibition reduces GO-induced caspase-3 activation and apoptotic signaling

**Fig. 2:**
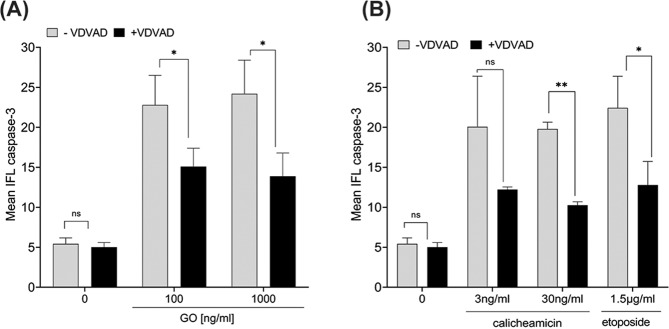
Reduced GO-, calicheamicin-, and etoposide-induced caspase-3 activation in AML cells upon caspase-2 blockage. **A**, **B** HL60 cells were exposed to GO-, calicheamicin- or etoposide for 48 h with or without pre-treatment with the caspase-2 inhibitor z-VDVAD-fmk (10 µM) for 2 h. The amount of active caspase-3 in HL60 cells was quantified by flow cytometry. Caspase-3 associated mean fluorescence intensity is presented. Histograms from one representative experiment showing activation of caspase-3 by a shift to the right is presented in Supplementary Fig. S1.

To further confirm if caspase-2 was required for GO-induced apoptosis, HL60 cells were incubated with the caspase-2 inhibitor z-VDVAD-fmk for 2 h prior to exposure of GO, calicheamicin- or etoposide treatment (Fig. 2A, B). In line with our previous results [13], GO-treatment caused a pronounced increase (4-fold higher level) in active caspase-3 compared to untreated cells (Fig. 2A, Supplementary Fig S1). Importantly, blocking the proteolytic activity of caspase-2 by z-VDVAD-fmk significantly reduced GO-induced caspase-3 activation by about 40% after either GO 100 ng/ml or GO 1000 ng/ml (*p* < 0.05 for both concentrations) (Fig. 2A, Supplementary Fig. S1). Similarly, calicheamicin-induced caspase-3 activation was clearly reduced in the presence of z-VDVAD-fmk, non-significantly at 3 ng/ml but with more than 50% inhibition at 30 ng/ml (*p* < 0.05), as was also the caspase-3 activation triggered by etoposide (*p* < 0.05) (Fig. 2B, Supplementary Fig. S1).

### Inhibition of caspase-2 does not block GO-induced pro-apoptotic conformational change in Bax but alters cleavage of full-length Bid

**Fig. 3:**
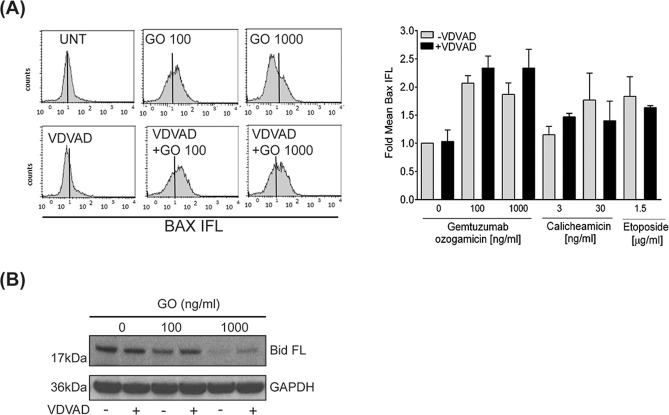
Caspase-2-mediated apoptotic signaling upon GO-treatment of AML cells does not involve conformational change of Bax. **A** Bax conformational change was studied in HL60 cells by flow cytometry after 48 h treatment with GO, calicheamicin or etoposide alone or combined with the caspase-2 inhibitor z-VDVAD-fmk (10 µM, 2 h pre-incubation)*. Left panel:* Bax conformational change is shown as a peak-shift to the right in the flow cytometry histogram. *Right panel*: Quantification of Bax-associated mean fluorescence intensity. Data is given as fold increase to untreated cells. **B** Cleavage of full-length Bid was analyzed by immunoblotting at 24 h post treatment with indicated doses of GO with or without 2 h pre-incubation with z-VDVAD-fmk (10 µM). GAPDH was used as loading control.

DNA damage-induced apoptosis may be preceded by pro-apoptotic conformational changes in the Bcl-2 family proteins Bax and Bak [33–35]. We earlier reported that efficient apoptosis induction after GO-treatment of AML cells requires such conformational changes in Bax and Bak [13]. Here we analyzed if activation of caspase-2 was required for conformational change in Bax upon GO-treatment (Fig. 3A). In line with our previous results [13], treatment with GO alone increased the amount of Bax, which showed a pro-apoptotic conformation by about 2-fold (Fig. 3A). Importantly, blocking caspase-2 proteolytic activity by z-VDVAD-fmk did not prevent such alteration in GO-induced Bax conformation which remain similar as when GO was used alone. Similarly, pre-incubation with z-VDVAD-fmk did not alter calicheamicin- or etoposide-induced Bax activation (Fig. 3A).

Caspase-2 has been shown to cleave Bid in response to ER-stress and blocking caspase-2 activity was reported to cause resistance to ER-stress-induced apoptosis [36]. Given these findings we analyzed if GO treatment altered Bid expression and if inhibition of caspase-2 proteolytic activity influenced this process (Fig. 3B). Treating HL60 cells with GO 100 ng/ml caused a slight decrease in full-length Bid expression while GO 1000 ng/ml clearly reduced expression of full-length Bid (Fig. 3B). Importantly, blocking caspase-2 proteolytic activity with z-VDVAD-fmk partially restored full-length Bid expression (Fig. 3B).

### Inhibition of caspase-2 protein expression blocks GO-induced activation of caspase-3 and PARP-1 cleavage

**Fig. 4:**
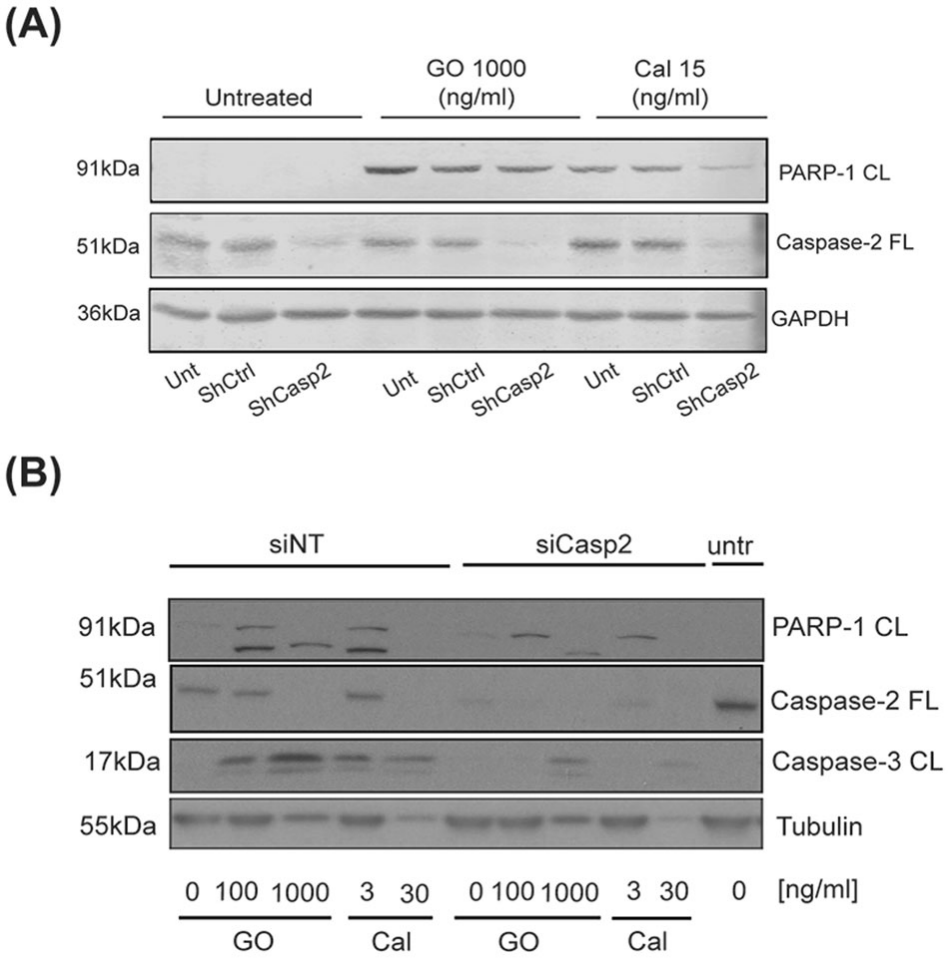
Inhibition of caspase-2 protein expression blocks caspase-3 activity and PARP-1 cleavage after GO-treatment. THP-1 AML cells were stable transduced with shRNA targeting caspase-2 (shCasp2) or empty vector (shCtrl) (**A**) or transiently transfected with siRNA targeting caspase-2 (siCasp2) or a non-targeting control sequence (siNT) (**B**). Cells were thereafter treated with either GO 100 ng/ml or 1000 ng/ml or calicheamicin 3 ng/ml, 15 ng/ml, or 30 ng/ml for 24 h (**A**) or 40 h (**B**). Full-length caspase-2, cleaved caspase-3, and cleaved PARP-1 protein expression were analysed by immunoblotting. α-Tubulin was used as loading control.

To further validate a role of caspase-2 in GO-induced apoptotic signalling, caspase-2 expression was ablated by shRNA or siRNA in AML THP-1 cells (Fig. 4). When comparing shRNA transduced cells to empty-vector transduced cells caspase-2 protein expression was clearly inhibited, as seen in lanes 3, 6, and 9 **(**Fig. 4A**)**. Similarly, a reduced expression of procaspase-2 was also evident when comparing siCasp2 treated samples to the siNT-treated or the untreated samples **(**Fig. 4B**)**. Treating the THP-1 cells with indicated doses of GO, or free calicheamicin resulted in dose-dependent cleavage of PARP-1 (Fig. 4A, B**)** and processing/activation of caspase-3 (Fig. 4B). Importantly, this cleavage of PARP-1 or activation of caspase-3 was partly inhibited in caspase-2 targeted cells (Fig. 4A, B), particularly at the lower doses of drugs (GO 100 ng/ml and calicheamicin 3 ng/ml), thus supporting our results based on caspase-2 inhibition by z-VDVAD (Fig. 2).

### Caspase-2 or caspase-3 expression at diagnosis does not correlate to outcome in AML patients

**Fig. 5:**
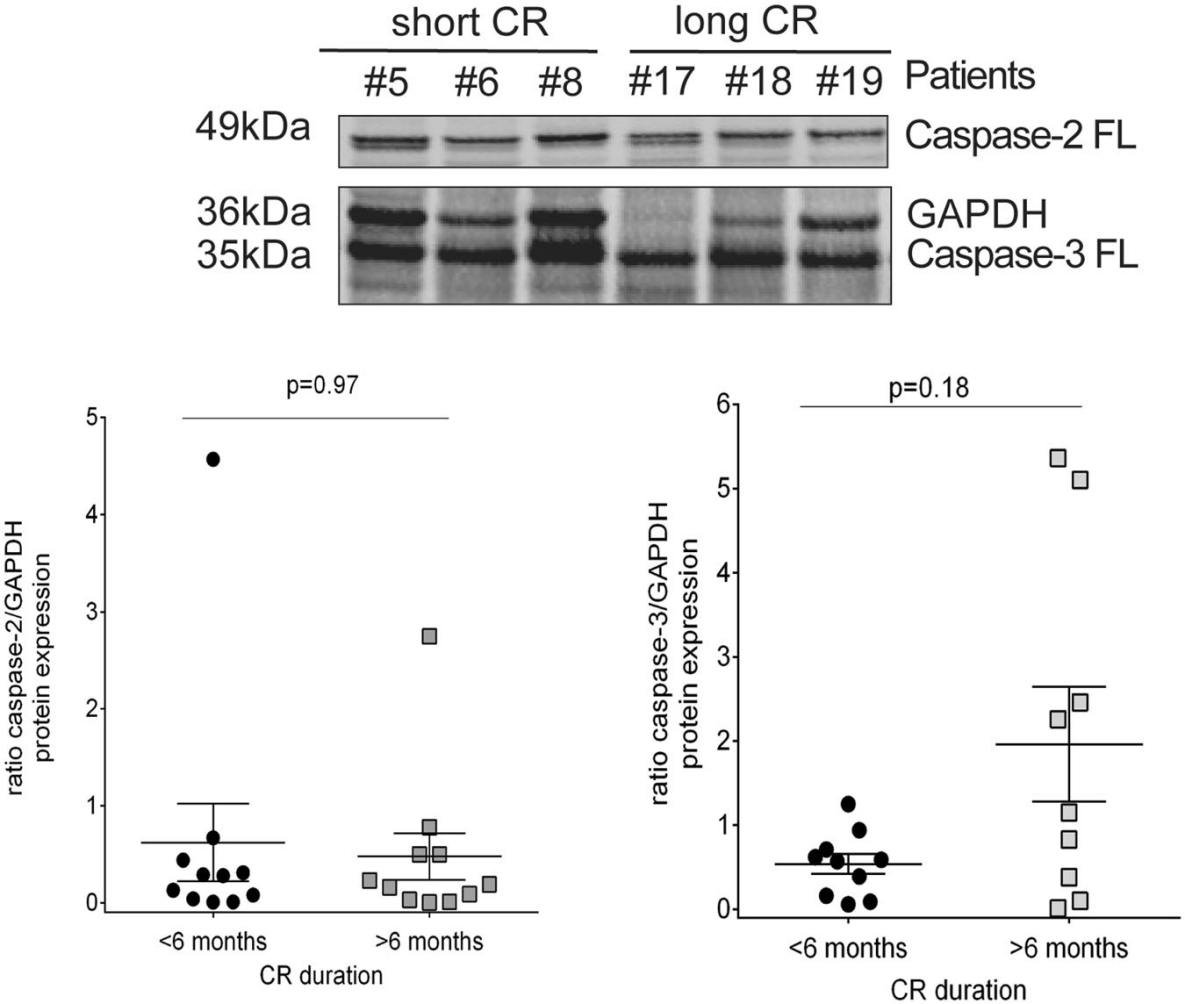
Basal caspase-2 and caspase-3 protein levels are heterogeneously expressed and do not correlate to complete remission duration in AML patients. Primary treatment naïve AML mononuclear cells (*n* = 22) were analysed for expression levels of full-length caspase-2 and full-length caspase-3 using immunoblotting. The samples illustrated in the top panel were all run on the same gel and the antibodies were run on the same membrane one at a time. Upper panel shows a immunoblot with full-length caspase-2 and full-length caspase-3 expressions in six patient samples with short (below 6 months) and long CR (above 6 months) respectively. GAPDH was used as loading control. Lower panels show the quantification of caspase-2 (**left**) or caspase-3 (**right**) expression levels in individual patients with short CR or long CR duration after normalization to GAPDH level. A two-tailed Mann- Whitney *t*-test was used for comparison of caspase-2 and caspase-3 expression levels in the two AML patient cohorts with p-values indicated.

Obtaining CR and its time of duration are important clinical endpoints in the outcome of AML patients. Earlier results concluded that caspase-3 expression and/or processing are associated to disease outcome in AML patients [37]. Given this and combined with our current results, which points towards a role of caspase-2 and caspase-3 in GO-induced apoptosis in AML cells, we next analysed whether full-length caspase-2 or caspase-3 expression levels prior treatment could be linked to CR duration in an AML patient cohort (n = 22) treated with conventional chemotherapy regiments. Full-length caspase-2 or caspase-3 expression levels were analysed in mononuclear cells isolated from AML patients at diagnosis, i.e., before treatment (for patient characteristics see Table 1). All patients were given induction chemotherapy with cytarabine and an anthracycline resulting in CR in all patients. The duration of CR was used to subdivide the patients into two groups: those with short (<6 months) and long (>6 months) CR, respectively. The expression of full-length caspase-2 and caspase-3 levels were analysed separately in these two patient groups, with some but not all patients analysed for both caspases (Fig. 5). The expression levels of either full-length caspase-2 or full-length caspase-3 varied considerably between individual patient samples after adjusting for western blot loading differences using GAPDH. However, no significant differences in the expression of either full-length caspase-2 (*p* = 0.97), or full-length caspase-3 (*p* = 0.18) was seen when comparing patients with long versus short CR duration.

**Table 1:** Characteristics of the AML patients.

	Patient	FAB	Cytogenetic analysis	CR (Days)
**Short CR (< 6months)**	#1	M2	No metaphases	79
	#2	M5A	46, XY, del (11)(q23) [25], 46, XY[10]	21
	#3	M1	46,XX [32]	12
	#4	M5B	46, XX [29]	38
	#5	M1	ND	26
	#6	M2	46, XX [43]	106
	#7	M1	46, XX [35]	113
	#8	M1	No metaphases	51
	#9	M2	46, XX	39
	#10	M1	46, XX [4]	109
	#11	M1	ND	56
**Long CR (> 6months)**	#12	M4	46, XY, inv (16)(p13q22)	304
	#13	M1	ND	235
	#14	M1	del (7q)(q22) [6]	588
	#15	M1	46, XX [29]	231
	#16	M1	t(8:21)(q22:q22), y- [21]	1182
	#17	M2	46, XX [22]	271
	#18	M1	46, XX, Ph + /46, XX, Ph + , -18, +mar/46, XX [29]	190
	#19	M2	47 [1]	714
	#20	M5B	ND	332
	#21	M5B	ND	893
	#22	M2	t(8:21)(q22:q22) [3] / 46, XY [9]	3701

*FAB* French–American–British classification of acute myeloid leukemia, *CR* complete remission duration, *ND* not determined (no sample analysed), No metaphases – no cytogenetic analysis of the sample possible; [x] – number of metaphases.

## Discussion

In this work, we demonstrate that the ADC gemtuzumab ozogamicin (GO; Mylotarg®) depends on caspase-2 for induction of apoptosis in AML cells and that the conventional drug etoposide similarly executes apoptosis in part via this route. Thus, GO caused caspase-2 processing in AML HL60 cells. GO also induced pro-caspase-2 processing in leukemic mononuclear cells from AML patients. Moreover, we found that chemical inhibition of caspase-2 proteolytic activity by the caspase-2 inhibitor VDVAD-fmk reduced GO-, calicheamicin- and etoposide-induced activity of caspase-3 and subsequent apoptosis. The activation of apoptotic signalling via processing of caspase-2 and caspase-3, as well as cleavage of PARP-1 was confirmed in THP-1 AML cells, where blocking caspase-2 expression by siRNA or shRNA reduced these GO-induced apoptotic events. We showed that GO-induced pro-apoptotic conformational change in Bax could proceed even upon inhibition of caspase-2 proteolytic activity while GO-induced Bid cleavage was reduced. Thus, our results suggest a role for caspase-2 in GO-induced apoptosis, but that certain GO-induced pro-apoptotic events commence via a caspase-2-independent route.

Calicheamicin, the active component of GO has previously been shown to trigger DNA DSBs formation [32] and GO is also reported to cause Chk1/Chk2 activation as part of its action mechanism [12]. Here we demonstrate that GO induced DNA DSBs formation at drug concentrations similar to the ones used for treating AML patients with GO [38]. When comparing calicheamicin-, etoposide- and GO-induced DNA DSBs formation, it was evident that the kinetics of GO-induced DNA DSBs was delayed relative to what was observed in response to the other agents. Such an effect is expected and can likely be explained by the fact that the GO complex needs to internalize after binding to CD33 and be further processed in the lysosomal compartment to allow free calicheamicin to diffuse into cell nuclei and target DNA, while the other agents directly may enter nucleus and trigger DNA DSBs formation.

Earlier results from us and others have demonstrated that caspase-2 plays a role in promoting and executing DNA damage-induced apoptosis in various tumor cell systems [14–17, 21, 24, 28–31]. For GO- and calicheamicin treatment, as well as etoposide, we also show a clear reduction in active caspase-3 when caspase-2 proteolytic activity was inhibited, thus suggesting that caspase-2 takes part in apoptosis execution in response to these agents in AML cells. To validate our findings obtained by inhibition of caspase-2 proteolytic activity using z-VDVAD-fmk we have applied transient caspase-2 siRNA transfection prior to GO-treatment in the same HL60 cells but failed to achieve stable ablation without inducing significant toxicity of the siRNA per se. We, therefore, used another AML cell line, THP-1 for knocking down the caspase-2 protein both transiently using siRNA and stably by shRNA methodologies. Ablating caspase-2 protein expression similarly as blocking its catalytic function resulted in reduced caspase-3 processing and PARP-1 cleavage after GO- and calicheamicin treatment in AML cells further supporting a role for caspase-2 in GO-induced apoptotic signaling.

In primary leukemic mononuclear cells from AML patients, GO treatment in vitro induced the disappearance of the 49 kDa full-length caspase-2 in GO-responsive patient cells but without detection of a cleaved 35 kDa caspase-2 fragment. One explanation might be that this caspase-2 fragment is rapidly further processed into the 12 kDa and 17 kDa subunits, which are the building blocks of the fully active enzyme [39].

We previously showed that failure to activate the mitochondria gatekeeper proteins Bak and Bax may contribute to the lack of active caspase-3 upon GO treatment [13]. The results presented in this study point towards a caspase-2-independent GO-induced Bax conformational change as activation was evident also when caspase-2 proteolytic activity was blocked.

The Bcl-2 family protein Bid may be cleaved as a result of apoptotic stimuli and the resulting truncated form, tBid has been demonstrated to control Bax complex formation and release of cytochrome *c* from mitochondria [40, 41]. Earlier, alteration of Bid level has been implicated in caspase-2 mediated apoptotic signaling in diverse cells including leukemic cells [42, 43]. Here we report that cleavage of full-length Bid upon GO treatment in AML cells is partly reduced by z-VDVAD-fmk treatment. The significance of the Bid cleavage in GO-induced cell death require, however, further analyses as the role of Bid in DNA damage signalling is conflicting [44–47].

The route to caspase-2 activation upon DNA damage may proceed via the PIDDosome or be independent thereof [19–23, 48, 49]. It has been reported that DNA damage may result in caspase-2 activation in the nucleolus in a complex with NPMI alongside PIDD and RAIDD [24]. The AML cells used in this study, HL60, as well as other GO-sensitive AML cells, are reported to be wild type for *NPM1* [50]. Hence, it is plausible that these cells may have NPMI in the nucleus allowing a capacity to trigger caspase-2 activation via such NPM1- complex in the nucleolus in response to the DNA damage inflicted by calicheamicin. Further analyses of both NPM1 and caspase-2 expression in nucleus prior and post GO treatment are therefore warranted.

One may hypothesize that the level of full-length caspase-2/-3 expression at time of AML diagnosis could represent a putative biomarker linked to subsequent treatment response. Indeed, Estrov et al reported that high levels of non-cleaved caspase-3, but low levels of the cleaved form, in diagnostic samples of AML were associated with poor patient survival [37]. The authors speculated that such high level of pro-caspase-3 could reflect a reduced ability of the leukemic cells to execute an apoptotic response upon chemotherapy challenge, possibly because of impaired PARP-1 cleavage and hence a still functional DNA-repair process. In our study, we failed to detect any significant differences of full-length caspase-3 expression in AML patients with long versus short CR duration at diagnosis. The discrepancy between our results and Estrov´s studies could be explained by the composition and the size of the AML patient cohorts analyzed. We did not either observe an association between full-length caspase-2 and clinical outcome, a finding in line with the data presented by Estrov et al. [37]. Nevertheless, further analyses of caspase-2 in AML cohorts are called for, especially in the light of NPM1 being part of a nucleolar route to caspase-2 activation [24, 51] and the *NPM1c* + mutation found in AML.

In conclusion, our results show that caspase-2 in part controls GO-induced apoptotic signaling in AML cells. Further analyses of caspase-2 in AML should be designed to elucidate whether restoration of caspase-2 apoptotic pathways may improve the response to chemotherapy and hence to advance the clinical outcome for patients with this disease.

## Materials and methods

### Cell culture and treatments

The majority of the experiments were carried out in the AML cell line HL60, isolated from a patient with acute promyelocytic leukemia (APL) [52]. HL60 cells were purchased from LGC Promochem AB tissue and cell culture tissue bank (Teddington, United Kingdom) and have previously been shown to express CD33 and to be sensitive to GO [12, 13]. The caspase-2 knockdown studies were carried out in the THP-1 human monocytic cell line derived from an AML patient (ATCC; LGC Standards, Wesel, Germany). All AML cells were maintained in RPMI-1640 medium supplemented with L-glutamine (2 mM) and heat-inactivated fetal calf serum (FCS, 10%; all from Sigma-Aldrich, Stockholm, Sweden).

Primary AML cells (Table 1) were isolated from peripheral blood of AML patients at diagnosis and prior to any treatment (obtained at Department of Hematology, Karolinska University Hospital, Stockholm, Sweden). The ethical permit was granted from the local ethical committee in Stockholm (Dnr.03-600 and 2007/1526-31/3) and written informed consent was obtained from all patients. Mononuclear cells (> 50% leukemic blast cells) were obtained from the samples using Ficoll Hypaque gradient isolation and were subsequently frozen in 50% serum and 20% dimethyl sulfoxide (DMSO). For in vitro experiments the primary mononuclear blast cells were thawed and maintained in RPMI-1640 medium with addition of 2mM L-glutamine, 10% FCS, granulocyte-macrophage colony-stimulating factor (GM-CSF, 10 ng/ml) and IL-3 (50 ng/ml) (all from Sigma-Aldrich).

A cohort of AML primary cells with long or short CR defined as <6 months or >6 months (Table 1) were used for western blot (WB) analyses of basal expression levels of full-length caspase-2 or caspase-3. In total, 22 patient samples were analysed on parallel gels. Caspase-2, caspase-3 and GAPDH were analysed on all gels. Samples that showed low quality on the WBs were excluded from the analyses. The characteristics of the patient samples included in the analyses are presented in Table 1.

Gemtuzumab ozogamicin (GO, Mylotarg) and calicheamicin were kind gifts from Wyeth-Ayers Research (Pearl River, NY). GO was dissolved in PBS (1 mg/ml) and calicheamicin in DMSO (0.6 mg/ml). Etoposide was purchased as ready-to-use infusions (Apoteket, Sweden).

### Cell viability analysis

To assess cell viability after treatment, cells were stained with 0.4% trypan blue in PBS (Sigma-Aldrich). The number of stained and non-stained cells in each sample was counted in a phase-contrast microscope and the number of viable (non-stained) cells after treatment was related to viable cells prior treatment (day 0), set to 100%.

### Apoptotic nuclear morphology analysis

For analyses of apoptotic nuclear morphology, cells were attached to glass slides by cytospinning, fixed in 0.25% formaldehyde (10% formalin solution, Bioreagens, Ellös, Sweden) in PBS and cell nuclei were stained using a 4’, 6-diamidino-2-phenylindole (DAPI)-containing mounting media (Sigma-Aldrich). Apoptotic morphology of cell nuclei was examined in a ZEISS Axioplan 2 imaging microscope with a Zeiss x63 lens with 200 cells calculated per sample and is given as percentage (%) of total cell nuclei assessed.

### Pulsed-field gel electrophoresis analysis of DNA double-strand breaks

Treatment-induced DNA DSBs were quantified by pulsed-field gel electrophoresis (PFGE), which allowed for separation of DNA fragments (size 1-10 Mbp) [53]. Briefly, HL60 cells were cultured in 1000 Bq/ml ^14^C-thymidine containing medium for 48 h prior to treatment. 1.2 × 10^6^ cells from each treatment were washed in ice-cold PBS, mixed with PBS and 1.2% low-melting agarose (ratio 1:1), and casted into plugs. DNA was extracted by incubating the plugs in lysis buffer (0.5 M EDTA (pH 8), 2% N-laurylsarcosine, 1 mg/ml proteinase K) for ≥20 h followed by immersion in high salt buffer (1.85 M NaCl, 0.15 M KCl, 5 mM MgCl_2_, 2 ml EDTA, 4 ml Tris, 0.5% Triton X-100 (pH 7.5); all chemicals were from Sigma-Aldrich) for 10 h. DNA treatment-induced DSBs were assessed by ^14^C scintillation counting of the gel that included a *Saccharomyces pombe* marker (Cambrex Bio Science Rockland, Inc., ME, US). The amount of DSBs is presented as fold increase in fragmented DNA (fragments < 5.7 Mbp) relative to untreated cells.

### Analysis of active caspase-3 and conformational change in Bax

Activation of caspase-3 or Bax conformational change occurring in response to treatment were examined in HL60 cells fixed in 0.5% formalin-solution as described [13]. Briefly, cells were permeabilized and stained by adding 100 µg/ml digitonin (Sigma-Aldrich) in PBS and further incubated with the fluorescein isothiocyanate (FITC)-conjugated antibody against the active form of caspase-3 (Cat no. 559341, BD Biosciences, Franklin Lakes, NJ, US) diluted 1:20 or the primary antibody targeting N-terminal conformational changed Bax (Cat no. 556467; BD Biosciences) diluted 1:250. For Bax staining an Alexa Fluor® 488 conjugated secondary antibody (dilution 1:in PBS, Cat no. A11001, Invitrogen, Carlsbad, CA, US) was used to reveal primary antibody binding. Fluorescent staining was assessed by flow cytometry in the FL-1 channel (FACS Calibur, BD Biosciences) and mean immunofluorescence intensity was quantified using the CellQuest Pro Software (BD Biosciences).

### Caspase-2 inhibition by chemical inhibitor or ablation of caspase-2 expression by siRNA or shRNA

To inhibit caspase-2 proteolytic activity, HL60 cells were pretreated for 2 h with z-VDVAD-fmk (10 µM, Cat no sc-3072, Santa Cruz Biotechnology, Santa Cruz, CA, US) after which GO, calicheamicin or etoposide were added. The inhibitor was present until cells were harvested for assessment of active caspase-3 or Bax conformational change by flow cytometry (see section above) or immunoblotting of cleaved caspase-3 or cleaved PARP-1 (see section immunoblot analysis below).

To transiently ablate caspase-2 expression THP-1 cells were transfected with ON-TARGET-plus SMARTpool siRNAs (Dharmacon, Lausanne, Switzerland). The siRNA against caspase-2 (siCasp2; L-003465-00) or a non-targeting sequence (siNT; D-001810-10) were mixed with transfection agent INTERFERin (Polyplus transfections, Illkirch, France) and added to the cells, according to manufacturer’s instructions. For stable transcriptional suppression of caspase-2, a pLKO.1-puro lentiviral shRNA plasmid (Open Biosystems/Thermo Fisher Scientific, Stockholm, Sweden) was used. Silencing of caspase-2 was achieved by the shCasp2 plasmid (TRCN0000003505/AAB70-D-8), and a non-target shRNA plasmid was used as a negative control (TRCN0000003505/AAB70-D-5; both from Open Biosystems Inc. Huntsville, Al, US). Replication incompetent viral particles was produced in low passage HEK293T packaging cells (kind gift from Prof. Galina Selivanova, Karolinska Institutet) by co-transfection with the compatible lentiviral packaging mix (Sigma-Aldrich), and by using the Lipofectamine 3000 transfection reagent (Thermo Fisher Scientific), according to the protocol recommended by the manufacturers. Lentiviral supernatants collected at 24 h and 48 h post-transfection were pooled, centrifuged (2000 rpm for 10 min at room temperature) and cleared through a 45 μm pore filter (VWR, Spånga, Sweden). Transductions of target cells were performed overnight without polybrene. The selection of shRNA-positive cells was accomplished by the addition of 2 μg/mL puromycin (Sigma-Aldrich) to growth media until non-transduced control cells were completely killed off (within 3-7 days). Knock of caspase-2 expression was confirmed by western blotting (see below).

### Immunoblot analysis

Proteins were extracted from whole cells cultured in vitro using RIPA buffer (50 mM Tris-HCl pH 7.4, 150 mM NaCl, 0.5% Igepal, 5 mM EDTA pH 8.0, 0.1% SDS) as described [13], or using cOmplete™ Lysis-M buffer complemented with Complete Protease Inhibitor Cocktail and PhosSTOP (Roche Diagnostics, Basel, Switzerland). 10-30 µg of total protein was separated in 3-8% Tris-acetate or 4-12% Bis-Tris NuPage gels (Invitrogen) and blotted onto nitrocellulose membranes (GE Healthcare, Uppsala, Sweden). Membranes were blocked in 5% bovine serum albumin (BSA) or Odyssey blocking buffer (LI-COR Biosciences, Bad Homburg, Germany) diluted 1:1 with TBST (0.5 M Tris, pH 7.6, 1.5 M NaCl, 0.05% Triton X-100). Primary antibodies targeting caspase-2 (#611023, Becton Dickinson, Franklin Lakes, NJ, US), caspase-3 (# 9662), cleaved caspase-3 (#9661), cleaved PARP-1 (#5625; all from Cell Signaling, distributer BioNordika, Stockholm, Sweden) and full-length Bid (ab2388, Abcam, Cambridge, UK) were used. To verify the loading of the samples an antibody against GAPDH (2275-PC-100, Trevigen, Gaithersburg, MD, US) or α-Tubulin (T5168, Sigma Aldrich) were applied. Fluorescent secondary donkey-anti-mouse- or goat anti-rabbit-antibodies, respectively (#926-32211 and #926-68072, LI-COR), were used to visualize primary antibody binding and signals recorded and analyzed on the Odyssey^®^ Sa Infrared Imaging System (LI-COR). In some experiments, the membranes were incubated with secondary goat anti-rabbit antibody and the proteins were visualized by the ECL + method (Pierce, Rockford, IL, US) with quantification carried out with the Quantity One software (Bio-Rad, Hercules, CA, US). Full blots of all western blots shown in this manuscript is found in Supplementary material 1–3. Presented protein bands are marked with squares.

### Statistical analysis

The cell viability, apoptotic assessment, PFGE, active caspase-3 and Bax conformational change experiments were performed in triplicates. Results are given as the mean ± standard deviation (S.D.). A two-tailed Mann-Whitney t-test was used for comparison of caspase-2 or caspase-3 expression levels in the two AML patient cohorts.

## Supplementary information

Supplemental Material

Supplementary Figures text

## Data Availability

All data generated and analysed during this study are included in this published article and supplementary information files.
