# Supplementary material for: Caspase-2 is a mediator of apoptotic signaling in response to gemtuzumab ozogamicin in acute myeloid leukemia
Source: Cell Death Discov. 2022 Jun 11;8:284. doi: 10.1038/s41420-022-01071-9 (PMC9188552; doi:10.1038/s41420-022-01071-9)

Supplemental material

Figure 1D

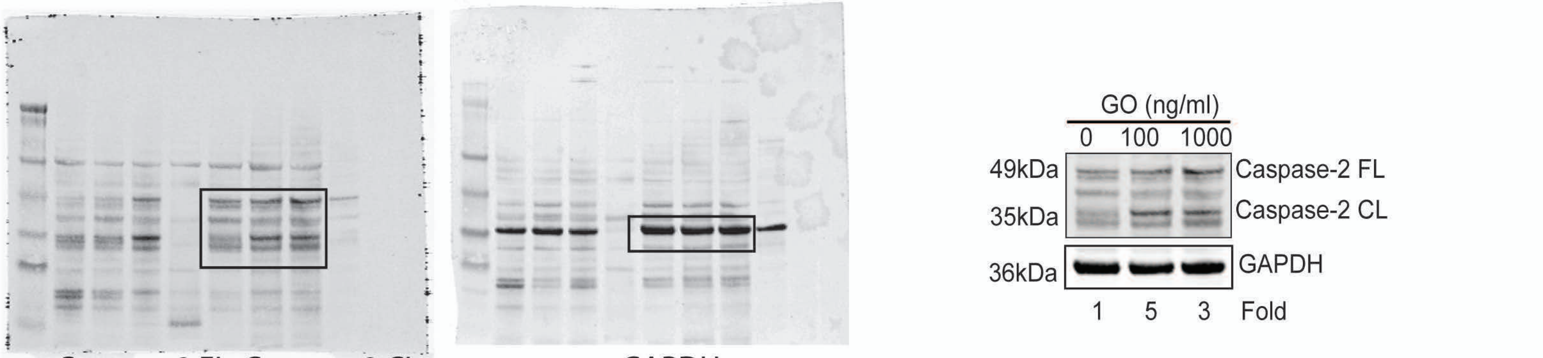

Caspase-2 FL, Caspase-2 CL

GAPDH

Figure 1E

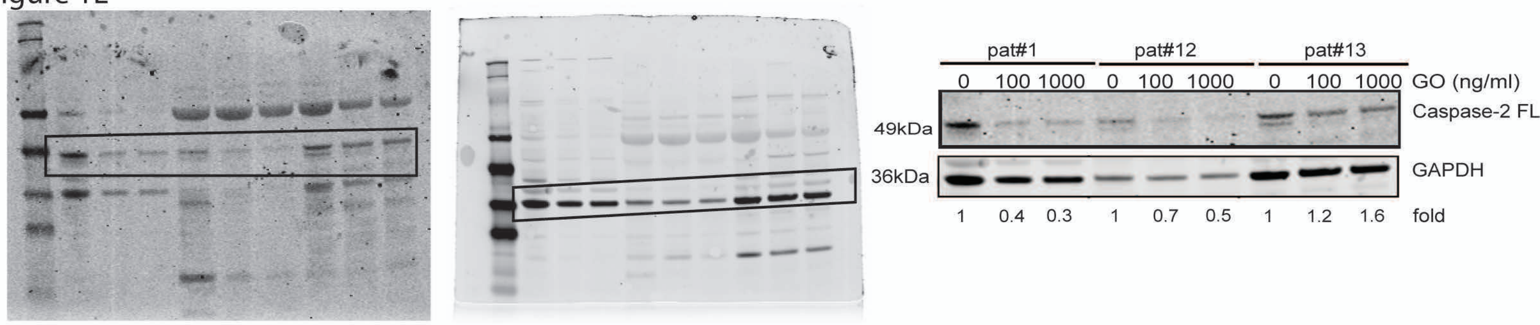

Caspase-2 FL

GAPDH

Figure 3C

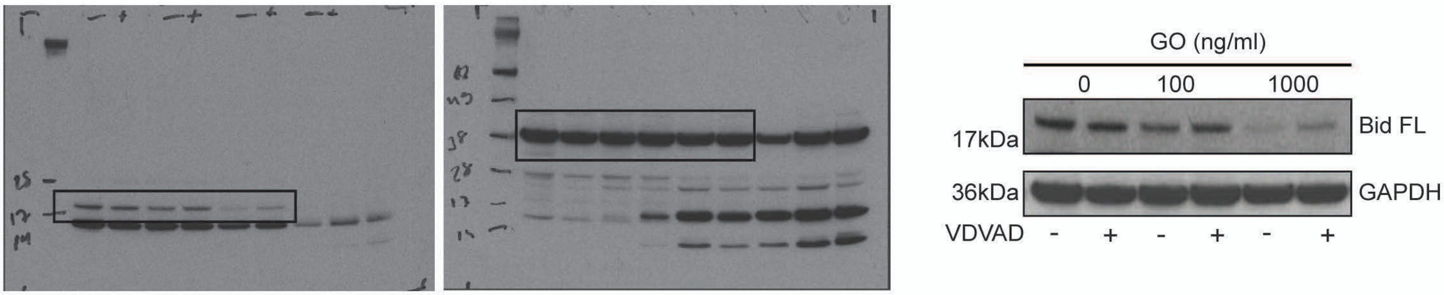

Bid FL

GAPDH

Figure 4A

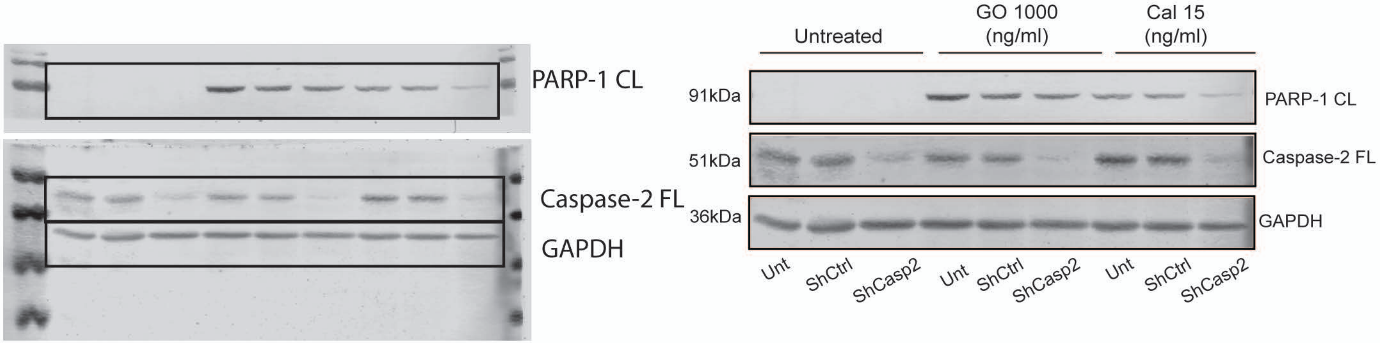

Figure 4B

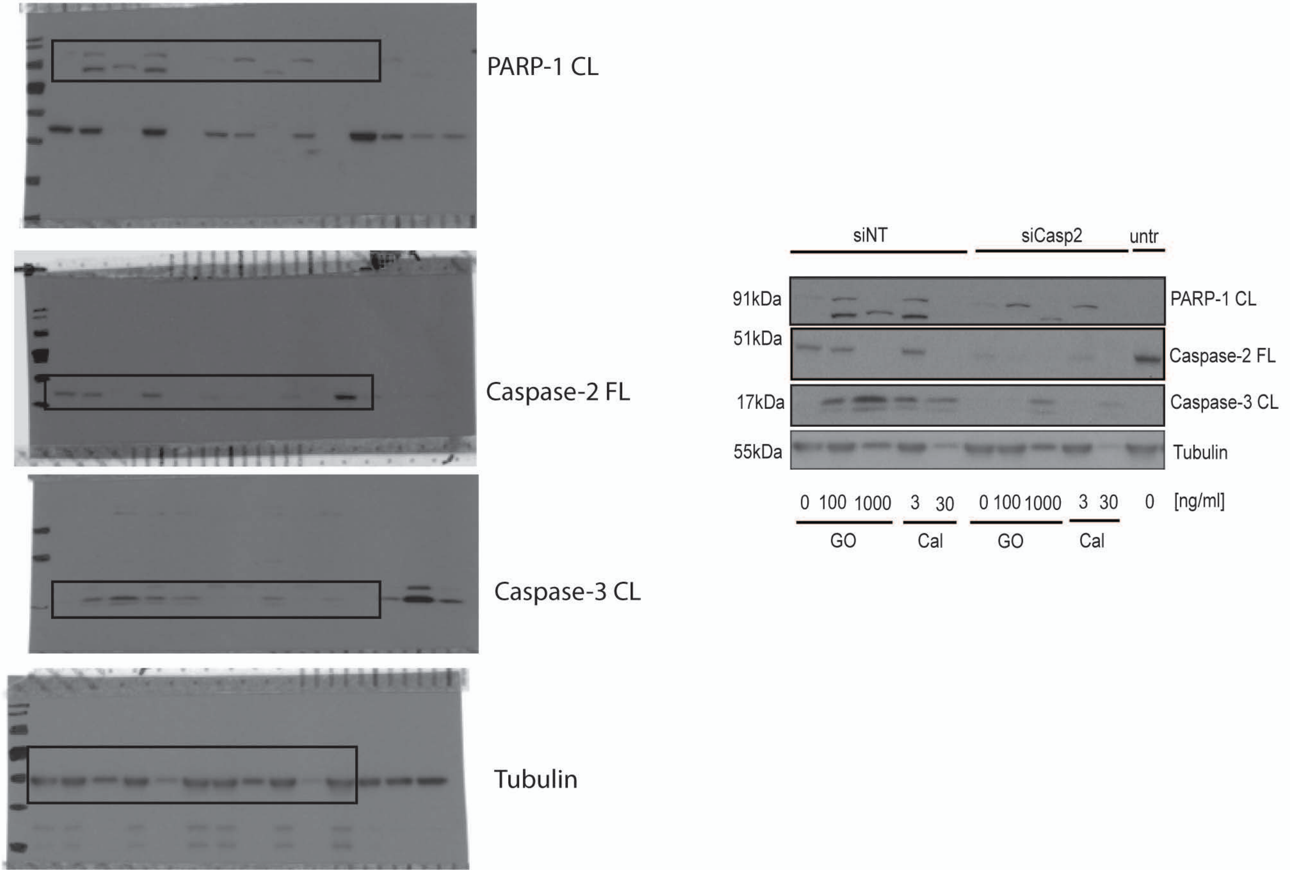

Supplemental material

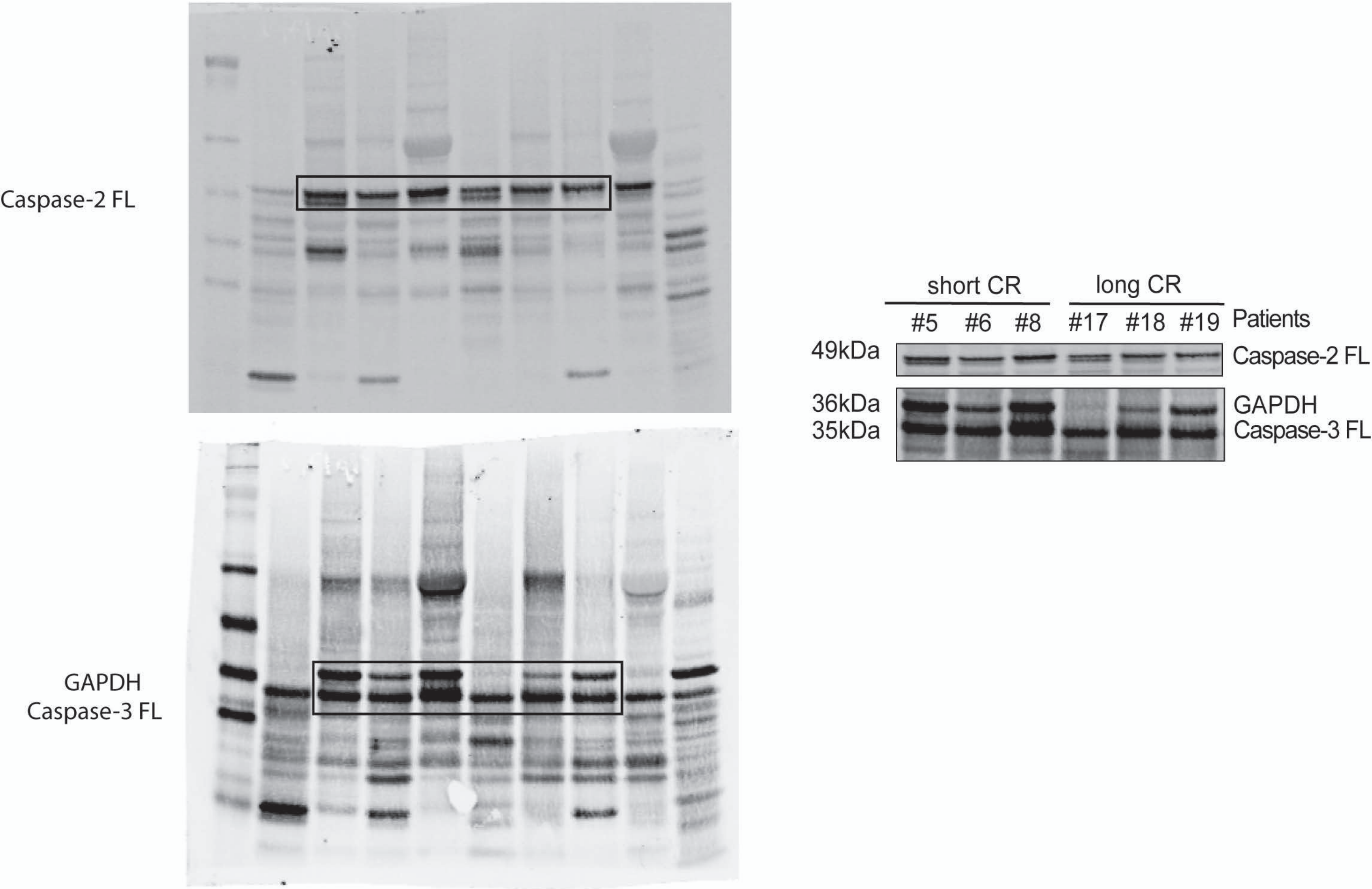

Supplement: Supplementary file 1 — Supplemental Material [file 41420_2022_1071_MOESM1_ESM.pdf]
