# Supplementary material for: Caspase-2 is a mediator of apoptotic signaling in response to gemtuzumab ozogamicin in acute myeloid leukemia
Source: Cell Death Discov. 2022 Jun 11;8:284. doi: 10.1038/s41420-022-01071-9 (PMC9188552; doi:10.1038/s41420-022-01071-9)
Supplement: Supplementary file 2 — Supplementary Figures text [file 41420_2022_1071_MOESM2_ESM.docx]

***Supplementary Figures:***

***Supplementary Figure S1. Inhibition of caspase-2 reduces GO-induced caspase-3 activation.*** HL60 cells were treated with GO-, calicheamicin- or etoposide for 48h with or without pre-treatment with 10µM caspase-2 inhibitor z-VDVAD-fmk for 2h. The effect on the amount of caspase-3 positive cells was examined by flow cytometry where caspase-3 active cells are presented with right shifted peak.

***Supplementary Material. Full membranes of all western blots.***

All western blots shown in the manuscript are here shown in their uncut, non-cropped versions. The cropped bands are marked on the full membranes and they are labelled as they are shown in the main paper.
